# The effect of a 6-month Schroth exercise program: a pilot study using subjects as their own controls

**DOI:** 10.1186/1748-7161-8-S2-O45

**Published:** 2013-09-18

**Authors:** Eric C Parent, Sanja Schreiber, Douglas Hedden, Marc Moreau, Douglas Hill, Elise Watkins

**Affiliations:** 1 University of Alberta, Alberta, Canada

## Background

Studies about Schroth exercises show that they may be effective in slowing curve progression and improving outcomes in individuals with adolescent idiopathic scoliosis (AIS)[1], however, these studies were of suboptimal quality. Systematic reviews highlight the need for randomized and prospective controlled studies on scoliosis-specific exercises [1].

## Purpose

The goal of this study was to determine the effect of Schroth exercises on curve progression using questionnaire scores of patients with AIS who were randomly assigned to spend six months as controls. 

## Methods

We included patients with AIS, 10-18 years, with curves of 10-45o, wearing a brace or not, and randomized initially into the standard-care group (observation or bracing) of an ongoing RCT. After receiving standard care for 6 months, controls received the 6-month Schroth intervention. Patients completed five introductory individual visits followed by 1-hour weekly supervised group sessions combined with daily home exercises prescribed using an algorithm (45 minutes). Outcomes were recorded at baseline, 6 and 12 months. Effect sizes were estimated using Cohen’s d (≥0.2=small, 0.5-0.8=moderate, >0.8=large), which corresponds to the difference between groups divided by the pooled standard deviation of the individual differences[2].

## Results

Of 13 subjects, 2 dropped out while controls and 2 while in Schroth therapy. Of the nine subjects who completed all follow-ups, the mean age and Cobb angle at baseline were 14.0±1.8yrs and 31±10.5o (17-43o), respectively. The recruitment rate was 14% among eligible participants, with time constraints limiting participation. All effect sizes favored Schroth except the Spinal Appearance Questionnaire (SAQ) waist score (0.14). Effect sizes for the SRS-22r questionnaire were as follows: self-image=0.92, pain=0.60, function=0.18 and total=0.56. Pain, self-image and total scores improvements were statistically significant (repeated measures ANOVA). SAQ effect sizes were as follows: general=0.23, chest=0.96, kyphosis=1.43, shoulders=0.40, trunk shift=0.44, prominence=0.77 and curve=0.89. Effect sizes for curve measures were as follows: major Cobb angle=0.00 and combined Cobb angles=0.13. Global ratings of change were significantly higher after Schroth therapy (4.7±2.3, 15-point scale). 

## Conclusions and discussion

All but one effect size favored Schroth and nearly half of the effect sizes were moderate or large. Of 70 patients, a change of 0.3-effect size would be considered to be a significant finding. Although only minor effects were observed on curve angles, Schroth exercises can positively affect appearance perception and quality of life, which are deemed priorities with scoliosis conservative treatments[3].

## References

[B1] Negrini S Fusco C Minozzi S Exercises reduce the progression rate of adolescent idiopathic scoliosis: results of a comprehensive systematic review of the literature Disabil.Rehabil 2008 30 772 85 10.1080/09638280801889568 18432435

[B2] Negrini S Grivas TB Kotwicki T Why do we treat adolescent idiopathic scoliosis? What we want to obtain and to avoid for our patients. SOSORT 2005 Consensus paper Scoliosis 2006 1 4 10.1186/1748-7161-1-4 16759352 PMC1475888

[B3] Cohen J Statistical Power Analysis for the Behavioral Sciences Lawrence Erlbaum Associates 1988 2 1 567

